# Identification of a Novel Hypovirulence-Inducing Hypovirus From *Alternaria alternata*

**DOI:** 10.3389/fmicb.2019.01076

**Published:** 2019-05-15

**Authors:** Huan Li, Ruiling Bian, Qian Liu, Liu Yang, Tianxing Pang, Lakha Salaipeth, Ida Bagus Andika, Hideki Kondo, Liying Sun

**Affiliations:** ^1^State Key Laboratory of Crop Stress Biology for Arid Areas and College of Plant Protection, Northwest A&F University, Yangling, China; ^2^School of Bioresources and Technology, King Mongkut’s University of Technology Thonburi, Bangkok, Thailand; ^3^Institute of Plant Science and Resources, Okayama University, Kurashiki, Japan

**Keywords:** mycovirus, hypovirus, *Alternaria alternata*, apple, leaf blotch disease, hypovirulence, defective RNA

## Abstract

Mycoviruses are wide spread throughout almost all groups of fungi but only a small number of mycoviruses can attenuate the growth and virulence of their fungal hosts. *Alternaria alternata* is an ascomycete fungus that causes leaf spot diseases on various crop plants. In this study, we identified a novel ssRNA mycovirus infecting an *A. alternata* f. sp. *mali* strain isolated from an apple orchard in China. Sequence analyses revealed that this virus is related to hypoviruses, in particular to Wuhan insect virus 14, an unclassified hypovirus identified from insect meta-transcriptomics, as well as other hypoviruses belonging to the genus *Hypovirus*, and therefore this virus is designed as Alternaria alternata hypovirus 1 (AaHV1). The genome of AaHV1 contains a single large open-reading frame encoding a putative polyprotein (∼479 kDa) with a cysteine proteinase-like and replication-associated domains. Curing AaHV1 from the fungal host strain indicated that the virus is responsible for the slow growth and reduced virulence of the host. AaHV1 defective RNA (D-RNA) with internal deletions emerging during fungal subcultures but the presence of D-RNA does not affect AaHV1 accumulation and pathogenicities. Moreover, AaHV1 could replicate and confer hypovirulence in *Botryosphaeria dothidea*, a fungal pathogen of apple white rot disease. This finding could facilitate better understanding of *A. alternata* pathogenicity and is relevant for development of biocontrol methods of fungal diseases.

## Introduction

Mycoviruses (fungal viruses) are widely distributed across all major groups of phytopathogenic fungi (Ghabrial and Suzuki, 2009; Pearson et al., 2009). Until recently, most reported mycoviruses were known to have double-stranded RNA (dsRNA) genomes, however, a number of single-strand RNA (ssRNA) mycoviruses have also been discovered (Ghabrial et al., 2015). Mycoviruses with ssRNA genomes are currently grouped into seven families: *Hypoviridae*, *Narnaviridae*, *Barnaviridae*, *Alphaflexiviridae*, *Gammaflexiviridae*, *Deltaflexiviridae* and *Mymonaviridae*, and one floating genus, *Botybirnavirus* (Amarasinghe et al., 2018; King et al., 2018), but many other mycoviruses have not been classified.

The family *Hypoviridae* contains a sole genus *Hypovirus* with four assigned virus species. Its members (Cryphonectria hypovirus 1–4, CHV1–4) infect a phytopathogenic fungus, *Cryphonectria parasitica*, the causal agent of chestnut blight disease (Suzuki et al., 2018). Hypoviruses are known as capsid-less viruses and they have large ssRNA genomes of 9.1–12.7 kb that possess either a single long open-reading frame (ORF) or two ORFs that encode polyproteins with a *cis*-acting papain-like cysteine protease at the N-terminus (Suzuki et al., 2018). In addition, many unclassified hypoviruses (or hypo-like viruses) have recently been discovered from other filamentous fungi, e.g., *Sclerotinia sclerotiorum* (Xie et al., 2011; Hu et al., 2014; Khalifa and Pearson, 2014; Marzano et al., 2015) *Valsa ceratosperma* (Yaegashi et al., 2012), *Fusarium graminearum* (Wang et al., 2013; Li et al., 2015), *Phomopsis longicolla* (Koloniuk et al., 2014), *Macrophomina phaseolina* (Marzano and Domier, 2016), *Botrytis cinerea* (Hao et al., 2018), and *Rosellinia necatrix* (Arjona-Lopez et al., 2018). Recent large-scale meta-transcriptomic analysis also uncovered that the hypo-like viruses are identified from non-fungal eukaryotes (invertebrates) (Shi et al., 2016). Based on phylogenetic analyses and genomic characteristics, CHV1–4 together with several unclassified hypoviruses were proposed to be classified into three genera, namely “Alphahypovirus” (including CHV1 and CHV2), “Betahypovirus” (including CHV3 and CHV4) and “Gammahypovirus” (Yaegashi et al., 2012; Hu et al., 2014; Khalifa and Pearson, 2014).

Mycoviruses are commonly associated with latent infections, while some are able to alter of fungal host phenotypes and/or attenuate the pathogenicity of fungal phytopathogenic hosts (Ghabrial and Suzuki, 2009; Pearson et al., 2009; Xie and Jiang, 2014). Mycovirus-associated hypovirulence has been studied extensively in CHV1-infected *C. parasitica* (Nuss, 2005; Eusebio-Cope et al., 2015; Rigling and Prospero, 2018). In addition, CHV2, CHV3, Botrytis cinerea hypovirus 1, Fusarium graminearum hypovirus 2 and the two strains of Sclerotinia sclerotiorum hypovirus 2 infections have also been associated with the hypovirulence of their fungal hosts (Hillman et al., 1994; Smart et al., 1999; Hu et al., 2014; Khalifa and Pearson, 2014; Li et al., 2015; Hao et al., 2018). In contrast, CHV4 and several other unclassified hypoviruses have no or a limited effect on fungal host virulence (Linder-Basso et al., 2005; Yaegashi et al., 2012; Wang et al., 2013; Koloniuk et al., 2014).

Apple leaf blotch disease caused by *Alternaria alternata* (family Pleosporaceae, class Dothideomycetes), has been a world-wide issue in apple production for decades (Johnson et al., 2000; Abe et al., 2010). *A. alternata* is generally considered a weak and opportunistic pathogen that infects a broad range of plants through various routes, such as wounds. Once infecting the plant, *A. alternata* is able to induce blackish spots on apple leaves in late spring or early summer, causing serious defoliation and declines in fruit quality (Filajdic and Sutton, 1995; Jung, 2007). The conventional use of fungicides to control apple leaf blotch disease is inefficient, therefore biocontrol methods are regarded as potential alternative means to control the disease. To date, several mycoviruses have been identified from *Alternaria* spp., including potential members of the genera *Chrysovirus*, *Partitivirus*, *Victorivirus*, *Botybirnavirus*, *Mitovirus*, and *Endornavirus* (Shang et al., 2015; Komatsu et al., 2016; Chen et al., 2017; Xiang et al., 2017; Okada et al., 2018; Xavier et al., 2018; Shamsi et al., 2019), as well as proposed the genus “Alternavirus,” family “Fusariviridae” and an undescribed novel taxon (Aoki et al., 2009; Lin et al., 2015; Zhong et al., 2016). It was reported that certain mycoviruses identified from *A. alternata* appear to impair the colony growth of their host fungus (Aoki et al., 2009; Fuke et al., 2011). Mycoviruses are also associated with the cyclic tetrapeptide tentoxin production in *A. alternata*, which causes chlorosis in seedlings of sensitive plants (Shepherd, 1988). Recently, Alternaria alternata chrysovirus 1 has been described to alter host fungus growth, while it also enhances the pathogenicity of fungal host on plants, most likely through the induction of host-specific toxin production (Okada et al., 2018). However, to date, no mycovirus has been reported to attenuate the pathogenicity of *Alternaria* spp.

In this study, we identified and characterized a novel hypovirulence-inducing hypovirus from *A. alternata* designated as Alternaria alternata hypovirus 1 (AaHV1). We also showed that AaHV1 produces defective RNA (D-RNA) during fungal culture in the laboratory. In addition, we demonstrated that AaHV1 confers hypovirulence in other plant phytogenic fungi.

## Materials and Methods

### Fungal Strains and Plant Materials

A total of 43 *A. alternata* strains were isolated from apple leaves with blotch disease randomly collected from a variety of apple orchards in Yangling County, Shaanxi Province of China in 2016. The *Botryosphaeria dothidea* strain, YL5, and *F. graminearum* strain, PH-1, were gifted from Dr. Zhonghua Ma (Zhejiang University, China) while the *C. parasitica* strain, EP155 (ATCC 3875) (Hillman et al., 1990) was a generous gift from Dr. Donald L. Nuss (University of Maryland). All fungal strains were maintained on potato dextrose agar (PDA) plates in the laboratory. For fungal identification, fungal DNA was isolated using standard phenol-chloroform extraction and ethanol precipitation, then used for polymerase chain reaction (PCR) amplification of the intergenic spacer (ITS) regions of ribosomal RNA gene (ITS1 and ITS2) (White et al., 1990). The amplified ITS sequences were subjected to a BLAST-N search.

All fungal strains were grown on PDA medium for 3–6 days at 24–26°C for morphological observation or on cellophane-covered PDA medium for RNA, DNA and protein extractions. Apple leaves (*Malus domestica* cv. Gala) subjected to pathogenicity tests were provided by Dr. Qingmei Guan (Northwest A&F University, China).

Fungus grew on different stress-inducing mediums for 5 days with the same growth condition as grown on PDA medium. The preparation of Vogel’s medium and stress-inducing mediums with the presence of 1 M NaCl, 1 M Sorbitol, 1% SDS and Congo red in PDA was described previously (Davis and de Serres, 1970; Zheng et al., 2012). All experiments used at least three independent fungal cultures.

### Fungal Inoculation

For inoculation of *A. alternata* with apple leaves, mycelia-containing gel plugs (around 0.5 × 1 cm), picked from the edge of a 3-day-old culture colony, were placed on newly grown apple leaves. The petioles of inoculated leaves were wrapped with water-wet cotton for moisturizing. Inoculated apple leaves were kept at 25°C, 70 to 80% humidity and with a photoperiod of 16 h/8 h (day/night). All inoculations were repeated three times.

### RNA Extraction, RT-PCR Detection and RNA Blot Analysis

Extraction of ssRNA and dsRNA from fungal mycelia followed the procedure described previously (Sun and Suzuki, 2008). DsRNA-enriched fraction was separated on 8% polyacrylamide gel electrophoresis (PAGE). For RT-PCR detection, first-strand cDNAs were synthesized using ReverTra Ace reverse transcriptase (Toyobo, Osaka, Japan) and amplified using 2 × mixture DNA polymerase (Kangwei, Beijing, China). For Northern blot analysis, Digoxigenin (DIG)-labeled DNA probes specific for genomic viral RNA (N: 180 to 900 nt; M: 7981 to 8603 nt and C: 13486 to 14039 nt, see Figure 4C) were utilized. The probes were prepared with the PCR DIG Probe Synthesis Kit (Roche Diagnostics, Penzberg, Germany). Gel electrophoresis and blotting were carried out as described previously (Andika et al., 2005). Hybridization conditions and detection of mRNAs were as described in the DIG Application Manual supplied by Roche. All of the primers used in this study are listed in Supplementary Table S1.

For viral dsRNA quantification, total RNA was prepared from *A. alternata* mycelia cultured on PDA covered with cellophane as described by Andika et al. (2017b). The final RNA concentration was adjusted to 0.1 μg/μl and used for agarose gel electrophoresis. The quantification of viral genomic dsRNA as described previously (Suzuki et al., 2003). Total RNA was extracted and subsequently electrophoresed in a 1.4% agarose gel in a 1 × TAE as described before (Sun et al., 2006). RNA bands were visualized with ChampGel 6000, a gel documentation and image analysis system (Beijing Sage Creation Science Co., Beijing, China). Relative amounts of viral genomic RNA were normalized to the amount of host fungal ribosomal RNA (rRNA).

### Next-Generation Sequencing and Rapid Amplification of cDNA Ends Analysis

The dsRNA-enriched fraction was used as a template for next-generation sequencing analysis. Preparation of the cDNA library was performed using NEBNext^®^ Ultra^TM^ RNA Library Prep Kit for Illumina (New England Biolabs Inc., Ipswich, MA, United States) and sequenced on the Illumina HiSeq 4000 platform (Illumina, San Diego, CA, United States). Raw reads were cleaned by removing adapter sequences, and low-quality bases (PHRED quality scores ≤ 5) were trimmed by a Trimmomatic package with default parameters so truncated reads smaller than 35 bp were discarded. All clean reads were then assembled through the *de novo* assembly program Trinity^1^ with a *K*-mer value = 25. Assembled reads were subjected to BLASTX searches with a cut-off of *E* ≤ 1e-5. To completely sequence the viral genome, RNA ligase-mediated (RLM)-rapid amplification of cDNA ends (RACE) was performed as previously described (Suzuki et al., 2004).

### Sequence and Phylogenetic Analyses

Sequence data were analyzed via GENETYX-MAC (Genetyx Co., Tokyo, Japan). Potential stem-loop RNA structures were predicted by employing Mfold version 2.3 (Zuker, 2003) (Web server URL:^2^). The conserved protein domains were predicted by the National Center for Biotechnology Information (NCBI) conserved domain database^3^. Putative transmembrane domains were determined using the TMHMM server version 2.0^4^ (Krogh et al., 2001). Multiple sequence alignments were performed using the MAFFT v7 (Katoh and Toh, 2008) or CLC Genomics Workbench v11 (CLC Bio-Qiagen, Aarhus, Denmark). Maximum likelihood (ML) phylogenetic tree construction was carried out as described previously (Kondo et al., 2015). Neighbor joining (NJ) (Saitou and Nei, 1987) tree was constructed based on the amino acid alignments using MAFFT. The trees were visualized with the FigTree 1.3.1^5^.

### Virus Transmission Assay

Hyphal anastomosis or hyphal fusion was conducted for viral horizontal transmission between fungal strains which are vegetatively compatible. Prior to hyphal fusion, the fungal partners were refreshed in PDA at 3 days. Co-culturing of fungal pairs was in 10-cm PDA plates in close proximity (10 mm) with each other and replicated across three plates. Mycelium blocks were taken randomly from the fungal pair, donor (virus source) side and recipient side (virus-free strain), to evaluate virus transmission. For viral vertical transmission, the assay was carried out to verify the frequencies of virus transmission through asexual spores. Fungal strains infected with the virus were cultured for 4 weeks on the bench top. Asexual spores were liberated in distilled water and spread on 10-cm PDA plates at appropriated dilutions. Single conidial germ lines were transferred to new PDA plates and cultured for 3 days. The number of infected colonies were scored based on visual observation of distinctive virus infection-associated colony morphologies and dsRNA or RT-PCR detection.

### Viral RNA Transfection

The polyethyleneglycol (PEG)-mediated transfection described by Sun et al. (2006) was performed. The purified total RNA containing viral RNAs was introduced into the freshly prepared protoplasts (Eusebio-Cope et al., 2009).

### Virulence Assay

The virulence of fungal colonies was assessed on apple leaves. Apple plants were generated from pathogen-free tissue culture and grown in a growth room. Fungi was inoculated on newly developed apple leaves and incubated on a bench top (25–27°C). Lesions were measured at days 5 and 7 post-inoculation. All inoculations were repeated three times.

## Results and Discussion

### Isolation of *A. alternata* Strains Carrying Virus-Like dsRNA Elements

**Figure 1:**
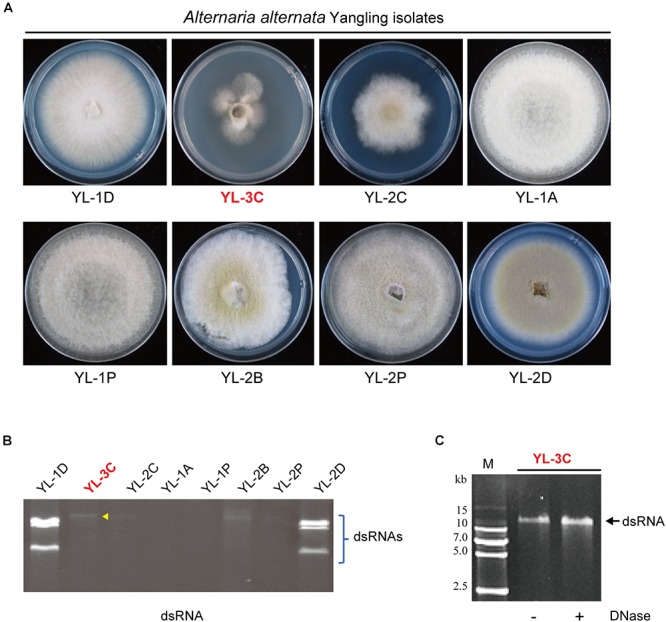
Isolation of *Alternaria alternata* strains carrying mycovirus-like double-stranded RNA (dsRNA) elements. **(A)** Phenotypic growth of *A. alternata* strains (Yangling isolates) on PDA medium. Colonies were grown on PDA for 5 days and photographed. **(B)** DsRNA profiles of the fungal strains shown in **(A)**. The entire gel image was provided in the Supplementary Figure S1B. The yellow arrow head shows the position of the band of a putative hypovirus. DsRNA samples were run on agarose gel and stained with EtBr. **(C)** Virus-like dsRNA element isolated from the YL-3C strain. After DNase treatment, the dsRNA sample was run on PAGE and stained. M, DNA size marker (DNA ladder VI, http://www.real-times.com.cn).

In order to study mycoviruses infecting *A. alternata*, 43 field fungal strains were collected from leaves of apple (*M. domestica* cv. Fuji) showing symptoms typical of Alternaria blotch disease (Supplementary Figure S1A). The collected fungal strains were isolated by hyphal tip culturing on PDA and re-inoculated to apple leaves to confirm that they are the causative agents of leaf blotch disease (Supplementary Figure S1A). Identification by sequencing with the ITS of rRNA with universal fungal primers for the ITS1 and ITS2 regions confirmed those isolates were *A. alternata* f. sp. *mali* (Woudenberg et al., 2015). To examine whether those field strains were carrying RNA mycoviruses, the presence of dsRNA was analyzed. Seventeen out of 43 fungal strains contained at least one dsRNA element but two strains (YL-2B and YL-5D2) lost dsRNA after subsequent subcultures on PDA (Figure 1A, Supplementary Figure S1B, and Supplementary Table S2). This suggest that those strains are infected with mycoviruses.

The phenotypic observation of mycelia growth of dsRNA-containing and dsRNA-free strains on PDA indicated a strain contained a large single dsRNA element (YL-3C strain) while also exhibiting slow growth (Figure 1A,B). PAGE analysis of purified dsRNA indicated that the dsRNA band present in YL-3C was around 14 kbp length and resistant against DNase treatment (Figure 1C). Thus, it is suggested that the presence of mycoviral-derived dsRNA in YL-3C attenuates the fungal growth.

### Identification of a Novel Hypovirus Infecting an *A. alternata* Strain

**Figure 2:**
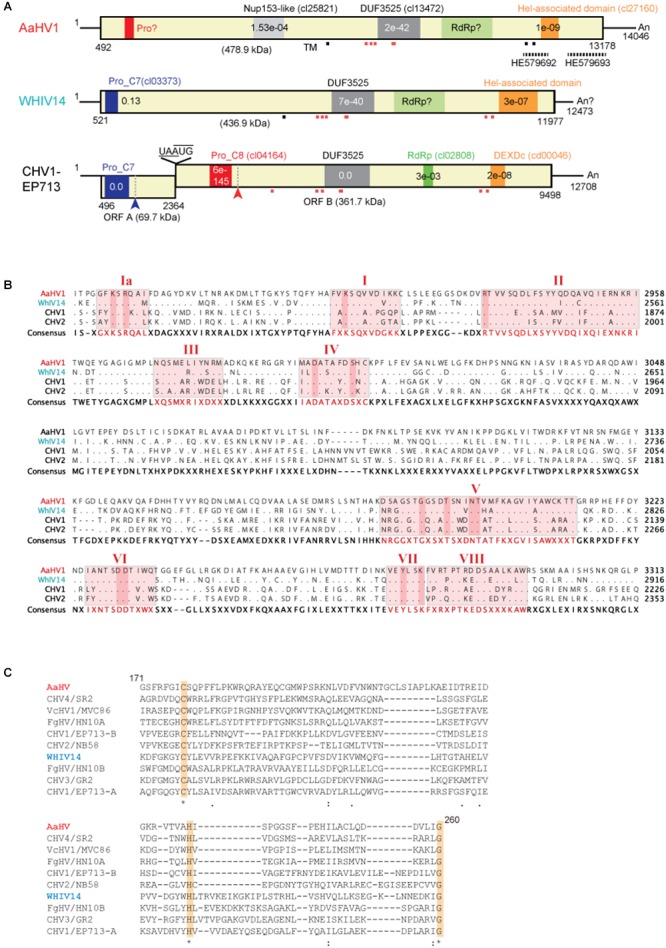
Genomic properties of Alternaria alternata hypovirus 1 (AaHV1). **(A)** Genome organization of AaHV1, Wuhan insect virus 14 (WhIV14) and Cryphonectria hypovirus 1 (CHV1). Dashed lines above HE579692 and HE579693 indicate the corresponding regions of similar virus-like sequences found in *A. alternata* pyrosequencing (Feldman et al., 2012). Small red or gray squares (strong and weak probabilities, respectively) indicate the position of predicted transmembrane domains (TM). **(B)** Amino acid sequence alignment of the region corresponding to RdRp domain. The position of nine core RdRp motifs and conserved residues (Koonin et al., 1991) were highlighted. **(C)** Amino acid sequence alignment of the region corresponding to cysteine protease. Conserved three cysteine protease core residues (cysteine, histidine, and glycine) (Koonin et al., 1991) are highlighted.

We determined the complete nucleotide sequence of the dsRNA molecule isolated from YL-3C using high-throughput sequencing and RACE methods. The full-length cDNA was 14,046 nucleotides (nts) in length consisting of a long ORF coding for a polypeptide of 4,228 amino acid (∼479 kDa) with a 5′-untranslated regions (UTR) of 491 nts, a 3′-UTR of 868 nts and an adenine tail (poly A) on the 3′-sequence end (Figure 2A). The sequence was submitted to GenBank with the accession number, MK189193. Based on BLAST-P analysis, the large viral-encoded protein has the highest identity (52%, coverage 74%, *E*-value = 0) with polyproteins encoded by Wuhan insect virus 14 (WhIV14), a hypo-like virus identified from insect meta-transcriptomics (Shi et al., 2016). Additionally, this viral protein has a similar identity (39–42%, coverage 50–64%, *E*-value = 0) to those of Macrophomina phaseolina hypovirus 1 (MpHV1) (Marzano et al., 2016), CHV1 (Shapira et al., 1991), Fusarium graminearum hypovirus 1 (FgHV1) (Wang et al., 2013) and CHV2 (Hillman et al., 1994; Supplementary Figure S2). Owing to its sequence relatedness to hypo- and hypo-like viruses, this newly identified virus is designated as Alternaria alternata hypovirus 1 (AaHV1). A BLAST-N search showed that AaHV1 has 81–85% identity homology with two hypovirus-like sequence fragments, 957 nts (GenBank accession HE579692) and 626 nts (GenBank accession HE579693), from *A. alternata* pyrosequencing (Feldman et al., 2012) (Figure 2A). By RT-PCR, two strains, YL-2C and YL-1E were found to contain AaHV1 RNA (Supplementary Table S2). AaHV1 disappeared in YL-2C after subsequent subcultures. YL-1E contains mixed virus infection so it is not further characterized in this study.

NCBI’s conserved domain search (Marchler-Bauer et al., 2017) showed that AaHV1-encoded proteins possess three conserved domains, Nucleoporin Nup153-like (accession cl25821), DUF3525 (accession cl13472) and helicase-associated domain (accession cl27160) with low or moderate *E*-values (Figure 2A). WhIV14 and CHV1 ORF B proteins also contain DUF3525 and helicase domains but the Nup153-like domain does not exist (Figure 2A). Notably, AaHV1 protein contains multiple transmembrane motifs around or within the DUF3525 and helicase domains, similar to the WhIV14 and CHV1 ORF B (Figure 2A). Unexpectedly, unlike in CHV1 ORF B protein, the RdRp domain was not detected in AaHV1 and WhIV14 proteins. However, AaHV1 and WhIV14 proteins were aligned with those of CHV1 ORF B and CHV2 ORF B proteins, in particular in the region between the DUF3525 and helicase domains where the presence of nine RdRp core motifs (Ia–VIII) (Koonin et al., 1991) were identified (Figure 2B). CHV1, which is the most well-studied hypovirus, processes viral polyproteins with two papain-like cysteine proteases encoded in ORF A and ORF B proteins (Choi et al., 1991; Koonin et al., 1991; Shapira and Nuss, 1991; Figure 2A). A papain-like cysteine protease motif was found in the N-terminal region of the WhIV14 protein but not in AaHV1 (Figure 2A). Nevertheless, alignment of the N-terminal region (171–260 aa region) of the AaHV1 protein with a papain-like cysteine protease domain regions of proteins encoded by other hypo- and hypo-like viruses uncovered the presence of conserved three cysteine protease core residues (cysteine, histidine and glycine in Figure 2C; Koonin et al., 1991), suggesting that AaHV1 encodes polyproteins processed by cysteine protease.

**Figure 3:**
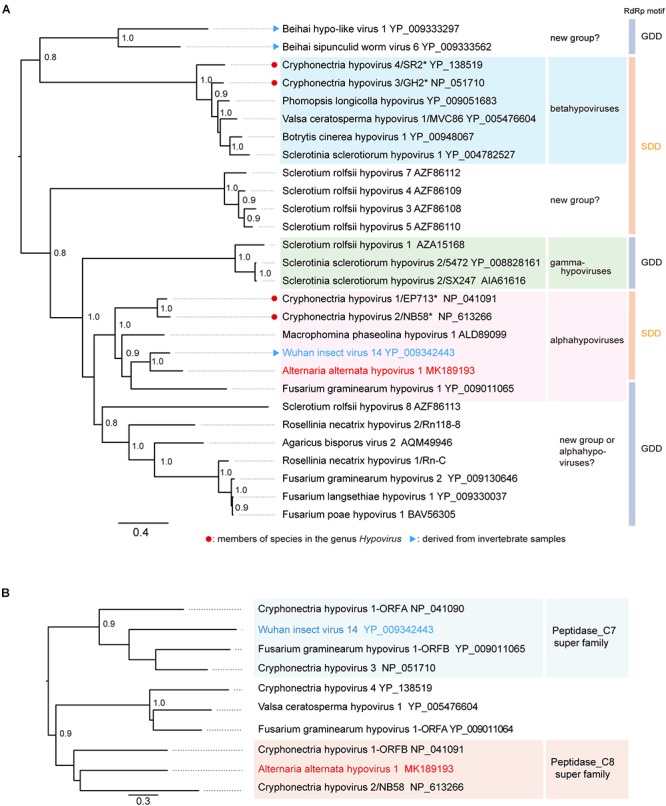
Phylogenetic relationships of AaHIV1 with other hypoviruses. **(A)** An ML phylogenetic tree based on the multiple amino acid sequence alignment of the replicase protein or its candidate protein sequences. Virus names and GenBank/Refseq accession numbers of replicase proteins are shown. The members of species for genus *Hypovirus* were shown with red circles. **(B)** An NJ tree based on cysteine protease or its candidate protein sequences. Numbers at the nodes indicate aLRT or bootstrap values (values less than 0.8 are not displayed).

The phylogenetic relationship of AaHV1 with other known hypo- and hypo-like viruses based on complete amino acid sequences of replication-associated proteins were analyzed according to the ML method. The results showed that AaHV1 was clustered with CHV1, CHV2, MpHV1, WhIV14, and FgHV1 (Figure 3A), which are the members of the proposed “Alphahypovirus” genus (Yaegashi et al., 2012). This proposed virus group has a conserved SDD tripeptide in RdRP motif IV (see Figure 2B), except for FgHV1, which is GDD. In most known (+) ssRNA viruses, the consensus tripeptide is GDD, while for segmented (-) ssRNA viruses and a limited number of (+) ssRNA viruses (coronaviruses), the commonly found consensus sequence is SDD (Poch et al., 1990; Xu et al., 2003). In addition, AaHV1 formed a clade with WhIV14 (Figure 3A), suggesting their close evolutionary relatedness. However, when the phylogenetic relationships were analyzed based on the regions corresponding to putative papain-like cysteine protease (Figure 2C), AaHV1 and WhIV14 were placed in separated clades, where AaHV1 protease-like domain was placed with CHV1 ORF B protease (p48, peptidase_C8 super family) while WhIV14 protease was found with CHV1 ORF A protease (p29, peptidase_C7 super family) (Figure 3B). Thus, although AaHV1 and WhIV14 generally maintain their close evolutionary relationship, they specifically diverge with respect to the type of papain-like cysteine protease. AaHV1 and WhIV14 possibly have different origins of their proteolytic domains owing to potential recombination(s) among ancestral hypoviruses, just as previously reported for modern CHV1 strains (Mlinarec et al., 2018).

The 5′- and 3′-UTRs are important for viral translation, replication and assembly (Newburn and White, 2015). AaHV1 and WhV14 5′-UTRs are not well conserved at nucleotide sequences (Supplementary Figure S3A). RNA secondary structure prediction showed the presence of multiple stem loops in the 5′-UTRs of AaHV1 and WhIV14, but the RNA conformations were different between those two viruses (Supplementary Figure S3B). Recently, the 5′-UTRs of hypoviruses (CHV1, CHV2, and CHV3) have been demonstrated to have an internal ribosomal entry site (IRES) function (Chiba et al., 2018), therefore it is of interest investigating whether the 5′-UTRs of AaHV1 and other hypo-like viruses, including WhIV14, have similar IRES activity.

### Molecular Characterization of AaHV1 Defective RNA

**Figure 4:**
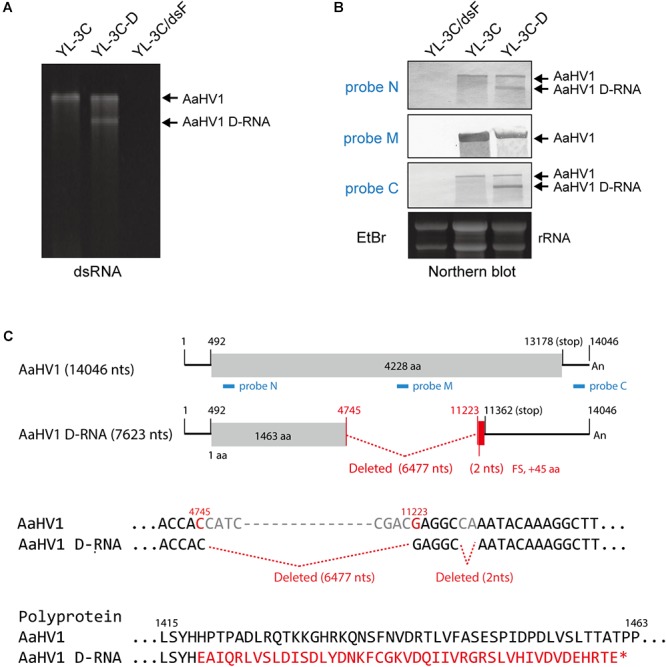
Identification of the defective RNA (D-RNA) of AaHV1. **(A)** DsRNA profiles of YL-3C with D-RNA (YL-3C-D). DsRNA samples were run on PAGE and stained with EtBr. **(B)** RNA blot analysis of YL-3C-D strain carrying AaHV1 D-RNA. The genomic position of the probes used for detection is shown in C (N, M and C, blue lines). **(C)** The sequence of AaHV1 D-RNA. The additional 30 aa owing to the frame shift in D-RNA is shown in red letters.

During subcultures of YL-3C isolate in PDA medium, YL-3C cultures were often found to contain an additionally a shorter dsRNA segment (namely YL-3C-D strain) (Figure 4A), suggesting the emergence of viral defective RNA or satellite-like RNA as previously observed for other hypo- or hypo-like viruses (Hillman et al., 2000; Zhang and Nuss, 2008; Xie et al., 2011; Li et al., 2015; Hao et al., 2018). To check this possibility, RNA blot analysis was carried out using three different probes specific for both terminal and middle portions of the AaHV1 genome (Figure 4C). The shorter RNA segment was detected through probes specific for both terminal regions but not the probe specific for middle region (Figure 4B). This result suggests that the shorter RNA segment is a D-RNA with internal genome deletion. Moreover, the presence of D-RNA does not affect the accumulation level of AaHV1 pertaining to the RNA genome (Figure 4B), indicating that the presence of D-RNA does not suppress AaHV1 replication, such as that commonly observed for viral defective interfering. The complete nucleotide sequence of AaHV1 D-RNA was determined. D-RNA has major (6,477 nts) and minor (2 nts) deletions in the central region, and these deletions render a truncated ORF with the addition of a novel 45 aa owing to the frame shift (Figure 4C). This result confirms that D-RNA is not a satellite-like RNA, with sequences that are only conserved at the 5′- and 3′-terminal regions and contain unrelated sequences. In the case of CHV1, D-RNA production requires the activity of the dicer-like 2 (DCL2) and argonaute-like 2 (AGL2) proteins, key components of antiviral RNA silencing pathways in filamentous fungus (Zhang and Nuss, 2008; Chiba and Suzuki, 2015). It is interesting to investigate whether RNA silencing is similarly essential for AaHV1 D-RNA production in *A. alternata*.

### Effects of AaHV1 Infection on Growth and Pathogenicity of *A. alternata*

**Figure 5:**
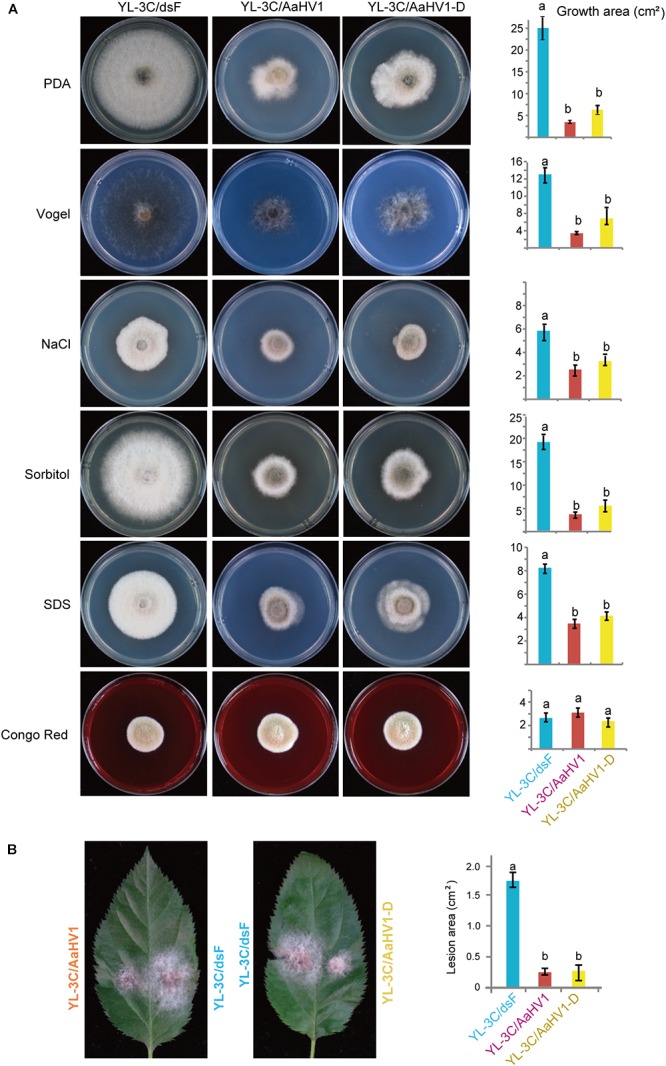
Effects of AaHV1 infection on fungal growth and virulence. **(A)** Phenotypic growth of AaHV1-free and -infected YL-3C strains (YL-3C/dsF, YL-3C, and YL-3C-D) on PDA and stress-inducing mediums. Colonies were grown on PDA for 5 days and photographed. Data are means ± SD (*n* = 3). Different letters indicate a significant difference at *p* < 0.01 (one-way analysis of variance (ANOVA) using MATLAB anova1 program). **(B)** The colonies of AaHV1-free and -infected YL-3C strains on apple leaves. Colonies were grown on leaves for 5 days and photographed. Data are means ± SD (*n* = 3). Different letter indicates a significant difference at *p* < 0.01 (one-way ANOVA).

AaHV1-free isogenic strain was obtained from YL-3C through single asexual spore isolation (YL-3C/dsF). We observed that AaHV1 was vertically transmitted through conidia with approximately 94–95% efficiency regardless of the presence of AaHV1 D-RNA (Supplementary Figure S4A). Moreover, from the AaHV1-infected parental strain (YL-3C), roughly 76% of the single spore isolates carried D-RNA (Supplementary Figure S4A), indicating the high emergence of D-RNA after vertical transmission through conidia. Both AaHV1 and AaHV1 + D-RNA were then re-introduced to the AaHV1-free isolate (YL-3C/dsF) through hyphal anastomosis to obtain YL-3C/AaHV1 and YL-3C/AaHV1-D (Supplementary Figure S4B). AaHV1-infected (YL-3C/AaHV1) and AaHV1 + AaHV1 D-RNA-infected (YL-3C/AaHV1-D) isolates clearly grew slower than AaHV1-free (YL-3C/dsF) isolates on PDA medium (Figure 5A). Accordingly, YL-3C/AaHV1 and YL-3C/AaHV1-D developed much smaller colonies than YL-3C/dsF on apple leaves (Figure 5B). This observation indicates that AaHV1 confers hypovirulence to the *A. alternata* strain, YL-3C, and the presence of D-RNA does not affect AaHV1’s ability to attenuate fungal virulence.

To obtain a general view surrounding what kind of stresses AaHV1 elicits in fungal hosts, we cultured YL-3C/dsF, YL-3C/AaHV1, and YL-3C/AaHV1-D on different stress-inducing mediums. The presence of 1 M NaCl or 1 M Sorbitol in PDA provides hyperosmotic stress conditions for fungal growth, while 1% SDS and Congo red treatment were used to mimic cytoplasmic membrane and cell-wall stresses (Zheng et al., 2012). Vogel’s medium was employed for the minimal medium for fungal growth (Davis and de Serres, 1970). Compared to YL-3C/dsF, YL-3C/AaHV1, and YL-3C/AaHV1-D showed obviously reduced mycelia growth across all tested fungal culture conditions except in Congo red treatment, where fungal growth was similar between AaHV1-free and -infected strains, indicating that AaHV1 was not able to induce relevant symptoms under Congo red treatment (Figure 5A). This result suggests that cytoplasmic membrane stresses may be connected with AaHV1 symptom induction on fungal hosts.

**Figure 6:**
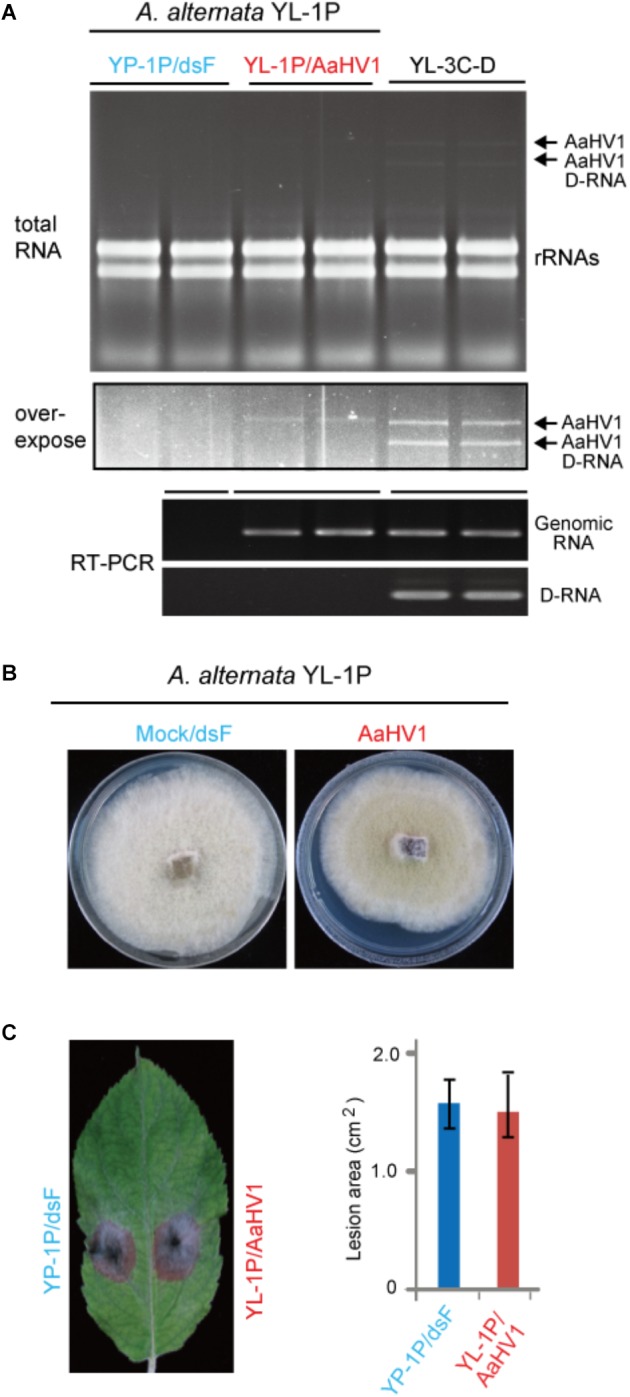
AaHV1 accumulation and pathogenicity in another *A. altenaria* strain YL-1P. **(A)** Relative AaHV1 dsRNA accumulation levels in the YL-1P and YL-3C strains. Total RNA samples were run on agarose gel and stained with EtBr. RT-PCR detection of AaHV1 using genome- and D-RNA-specific primer sets (Supplementary Table S1). **(B)** Phenotypic growth of AaHV1-free and -infected YL-1P strains on PDA mediums. Colonies were grown on PDA for 5 days and photographed. **(C)** The colonies of AaHV1-free and -infected YL-1P strains on apple leaves. Colonies were grown on leaves for 5 days and photographed. Data are means ± SD (*n* = 3).

To investigate AaHV1 pathogenicity in different *A. alternata* host backgrounds, we introduced AaHV1 into a YL-1P strain that contains no detectable dsRNA (Figure 1A). Sequencing the ITS1 and ITS2 regions revealed this strain is also *A. alternata* f. sp. *mali*. Co-culture experiments demonstrated that YL-1P and YL-3C isolates belong to different vegetative compatibility groups and thus AaHV1 was not horizontally transmitted to YL-1P by hyphal anastomosis (data not shown). We then introduced AaHV1 to YL-1P by spheroplast transfection with total RNA isolated from YL-3C mycelia. The conidial isolates from AaHV1-transfected YL-1P were re-generated and subjected to four rounds of repeated subculture on PDA plates. It is interesting that all AaHV1 detected in the YL-1P host background did not produce D-RNA (data not shown). This result was consistently confirmed after further subculture (six rounds). As seen in Figure 6A, AaHV1 genomic dsRNA accumulated at lower levels in YL-1P than the YL-3C-D strain. RT-PCR with primer sets specific for the 5′ terminal of genomic RNA and the junction sequence in the D-RNA (Supplementary Table S1) was employed to further confirm the infection of YL-1P with AaHV1 (Figure 6A). D-RNA was only detected in the YL-3C strain, confirming that no D-RNA was accumulated in the YL-1P strain. Low accumulation of AaHV1 in the YL-1P strain may be associated with its genome stability.

AaHV1-infected YL-1P grew slightly slower than dsRNA-free YL-1P (Figure 6B). Moreover, AaHV1-free and -infected YL-1P strains developed similar size of lesions on apple leaves (Figure 6C), indicating that AaHV1 does not confer hypovirulence to the YL-1P strain. These observations suggest that AaHV1 pathogenicity differs among different *A. alternata* f. sp. *mali* strains. Moreover, AaHV1 accumulation is lower in YL-1P than YL-3C strains, suggesting that different *A. alternata* strains have varying defense strengths against virus infection.

### AaHV1 Infectivity and Pathogenicity in Other Fungal Species

**Figure 7:**
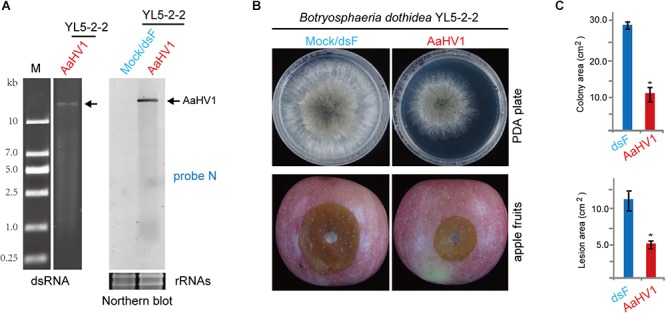
AaHV1 infectivity and pathogenicity in *Botryosphaeria dothidea*. **(A)** AaHV1 RNA in *B. dothidea* YL5 strain (YL5-2-2 isolate) detected by dsRNA isolation and RNA blotting. **(B)** Phenotypic growth and virulence of AaHV1-free and -infected *B. dothidea* strains on PDA medium and apples. Colonies were grown on PDA (for 4 days) and leaves (for 4 days) and photographed. **(C)** The lesion area on apples described in B. Data are means ± SD (*n* = 3). Asterisk indicates *p* < 0.01 (Student’s *t*-test).

To assess AaHV1 infectivity in other Ascomycota fungal species, AaHV1 was introduced (transfected) to *B. dothidea*, *F. graminearum* and *C. parasitica*, which are the fungal pathogens of apple white rot (or ring rot), wheat head blight and chestnut blight diseases, respectively (Vasilyeva and Kim, 2000; Tang et al., 2012; Rigling and Prospero, 2018). AaHV1 accumulation was detected in *B. dothidea* (family *Botryosphaeriaceae*, belonging the same class Dothideomycetes with *Alternaria* spp.), but not in *F. graminearum* PH-1 (family *Nectriaceae*, class Sordariomycetes) and *C. parasitica* EP155 (family *Cryphonectriaceae*, class Sordariomycetes) (Figure 7A and data not shown). It is interesting to note that AaHV1 infection markedly reduced *B. dothidea* growth on PDA medium and apples (Figure 7B,C). At present, four RNA mycoviruses are known to infect the *B. dothidea* strains, two of them (a chrysovirus and proposed polymycovirus) are associated with conferring hypovirulence-associated traits to fungal hosts (Wang et al., 2014; Zhai et al., 2015, 2016). Our data indicates that AaHV1 could confer hypovirulence in a heterologous fungal host (unnatural host). This observation also implies that AaHV1 has potential for use as a biocontrol agent for other fungal crop diseases.

## Conclusion

In this study, we characterized a novel mycovirus related to members of the *Hypoviridae* family from a phytopathogenic fungus, *A. alternata*. Although a number of mycoviruses have been identified from *A. alternata*, this is the first report of a hypovirulence-inducing mycovirus in *A. alternata*. Therefore, the finding with this virus provides a valuable experimental system to study the molecular aspects of virus-fungus interactions and fungal pathogenicity in *A. alternata*. Hypoviruses have a prominent place in mycovirology, in particular owing to long and intensive studies on CHV1 in the fungal host, *C. parasitica* (Nuss, 2005). CHV1 provides the first example of the successful use of a mycovirus as a biocontrol agent (Rigling and Prospero, 2018). In addition, many molecular aspects of mycovirology, including replication, pathogenicity and RNA silencing-associated host immunity, have been elucidated utilizing the CHV1-*C. parasitica* pathosystem (Zhang and Nuss, 2008; Chiba and Suzuki, 2015; Eusebio-Cope et al., 2015; Andika et al., 2017a, 2019). Like CHV1, AaHV1 also confers strong hypovirulence to fungal hosts and possess similar molecular properties. In this regard, AaHV1 is also a prospective research material for further study of the fundamental and applicative aspects of mycovirology.

## Data Availability

No datasets were generated or analyzed for this study.

## Author Contributions

LSu designed the experiments. HL, RB, QL, LY, TP, and LSa performed the experimental work. IBA, HK, and LSu analyzed the data and wrote the manuscript.

## Conflict of Interest Statement

The authors declare that the research was conducted in the absence of any commercial or financial relationships that could be construed as a potential conflict of interest.

## Footnotes

1 http://trinityrnaseq.github.io

2 http://mfold.rna.albany.edu/

3 https://blast.ncbi.nlm.nih.gov/Blast.cgi

4 http://www.cbs.dtu.dk/services/TMHMM/

5 http://tree.bio.ed.ac.uk/software/

## Supplementary Material

FIGURE S1 **(A)** *Alternaria alternata* lesion on apple leaves after isolation and re-inoculation. **(B)** DsRNA profiles of *A. alternata* strains isolated from apple leaves.

FIGURE S2 BLAST-P result using the AaHV1-encoded protein as a query.

FIGURE S3 **(A)** Alignment of 5′-UTR sequences of AaHV1 and WhIV14. The putative start codons are highlighted with red color. **(B)** Predicted RNA secondary structures of 5′-UTR sequences of AaHV1 and WhIV14 (not shown the entire 5′-UTR regions).

FIGURE S4 **(A)** Efficiency of vertical transmission of AaHV1 through conidia with or without its D-RNA element (YL-3C-D and YL-3C, respectively). **(B)** Horizontal transmission of AaHV1 and its D-RNA element through hyphal fusion. The dots were the position where mycelia took for sub-culturing.

TABLE S1 A list of primers used in this study.

TABLE S2 Detection of AaHV1 in 43 *Alternaria alternata* strains.
