# Supplementary figures and images for: Identification of a Novel Hypovirulence-Inducing Hypovirus From Alternaria alternata
Source: Front Microbiol. 2019 May 15;10:1076. doi: 10.3389/fmicb.2019.01076 (PMC6530530; doi:10.3389/fmicb.2019.01076)

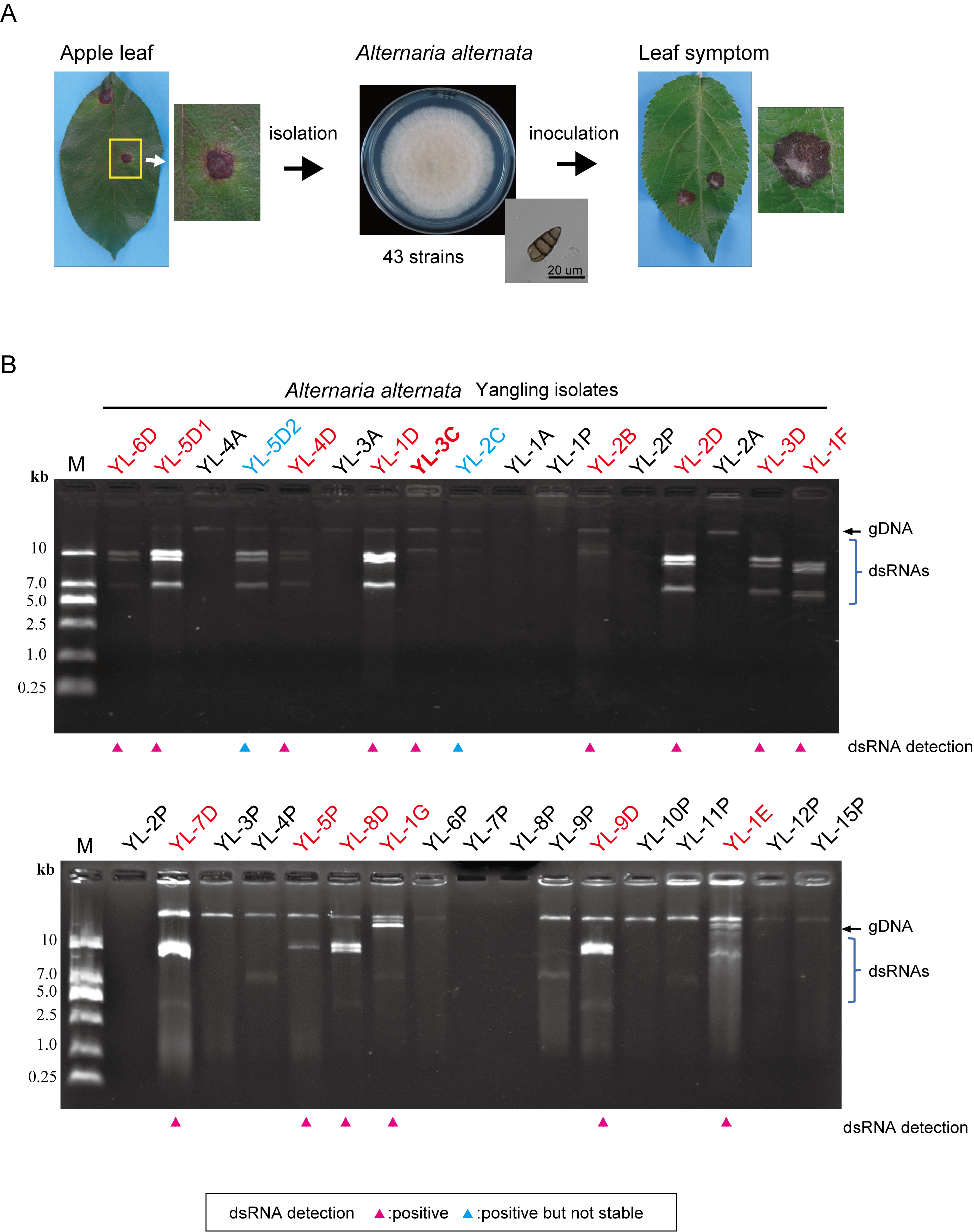

Supplement: FIGURE S1 — (A) Alternaria alternata lesion on apple leaves after isolation and re-inoculation. (B) DsRNA profiles of A. alternata strains isolated from apple leaves. [file Image_1.tif]

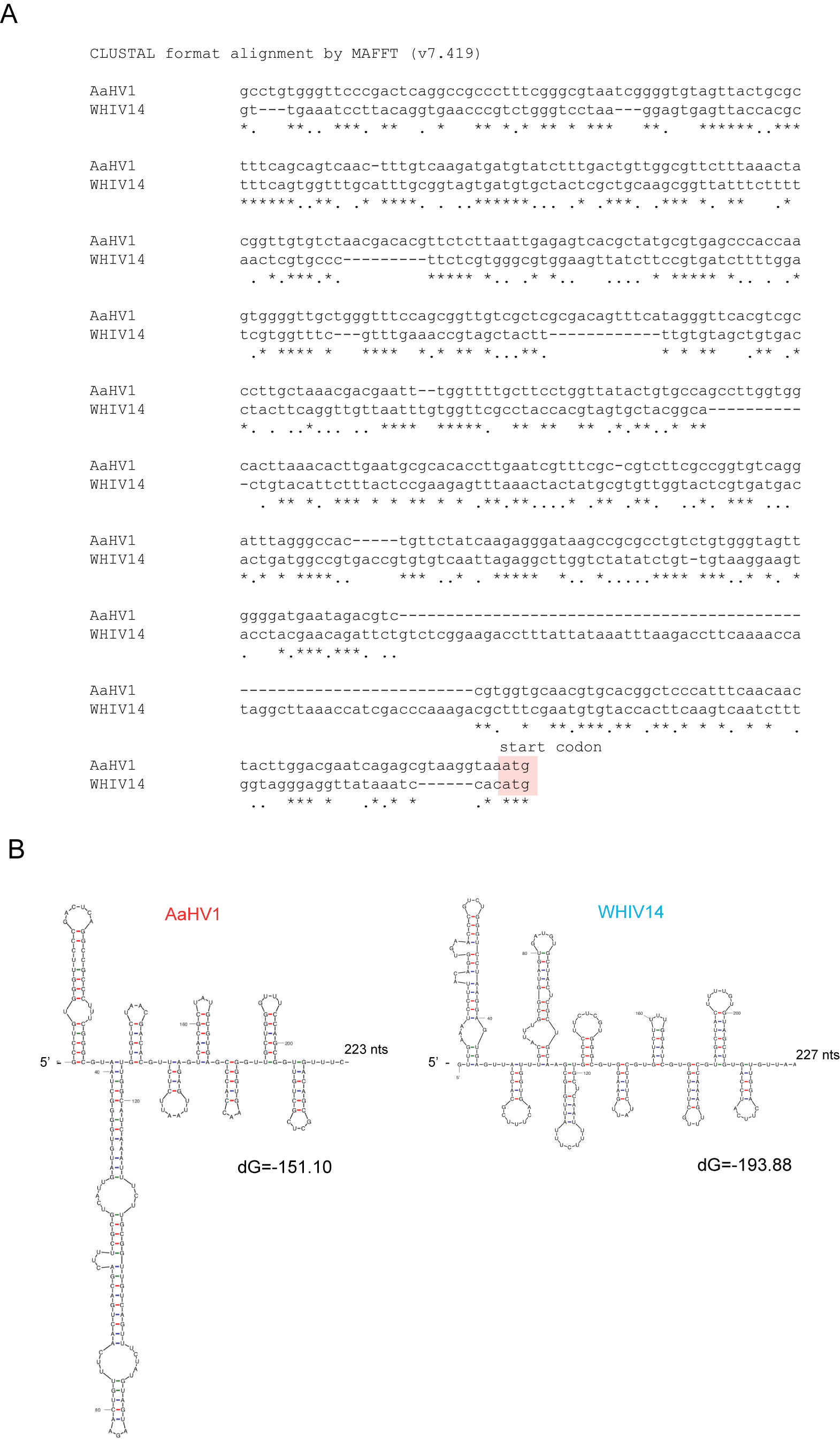

Supplement: FIGURE S3 — (A) Alignment of 5′-UTR sequences of AaHV1 and WhIV14. The putative start codons are highlighted with red color. (B) Predicted RNA secondary structures of 5′-UTR sequences of AaHV1 and WhIV14 (not shown the entire 5′-UTR regions). [file Image_3.tif]

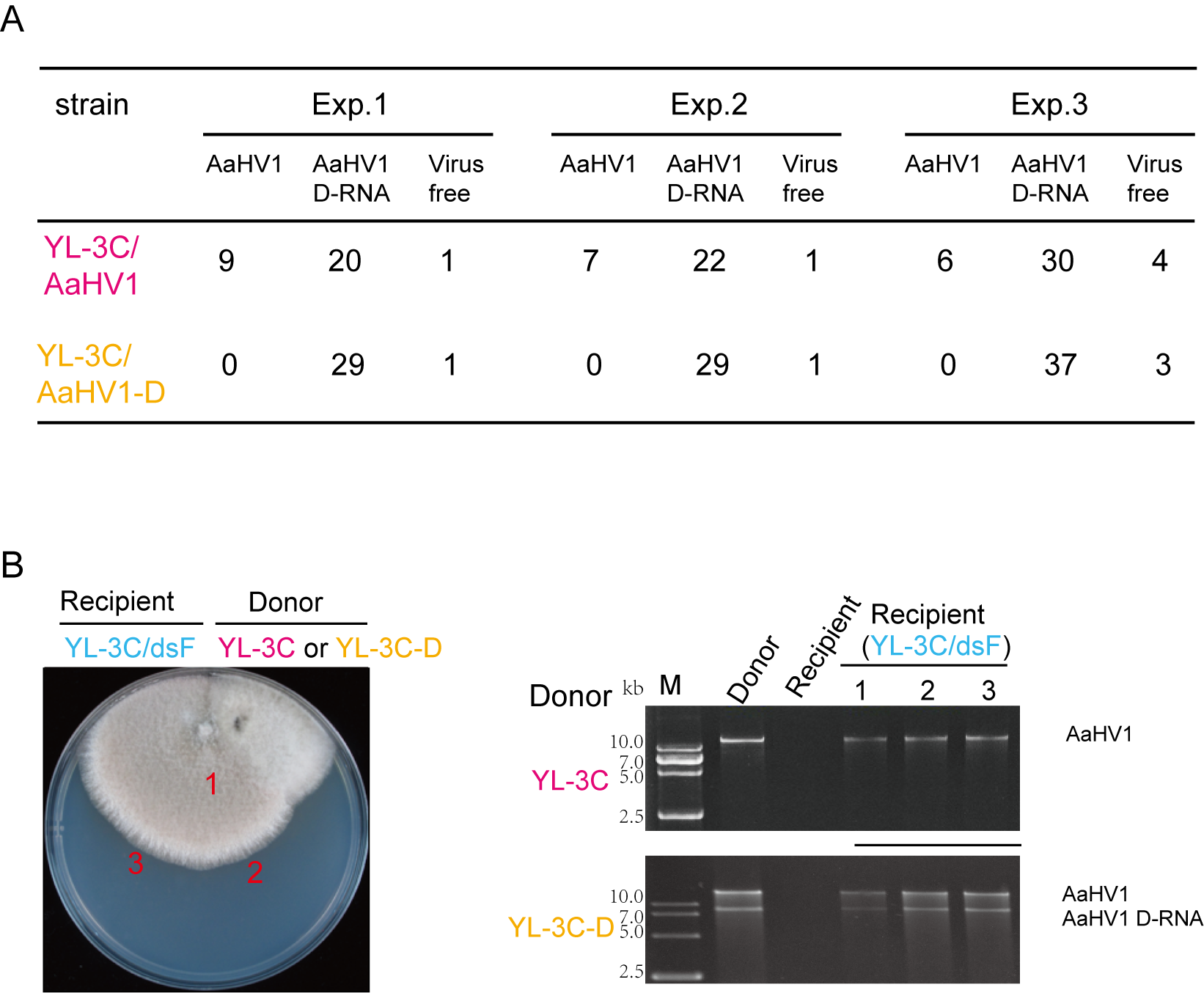

Supplement: FIGURE S4 — (A) Efficiency of vertical transmission of AaHV1 through conidia with or without its D-RNA element (YL-3C-D and YL-3C, respectively). (B) Horizontal transmission of AaHV1 and its D-RNA element through hyphal fusion. The dots were the position where mycelia took for sub-culturing. [file Image_4.tif]
