# Supplementary material for: Identification of a Novel Hypovirulence-Inducing Hypovirus From Alternaria alternata
Source: Front Microbiol. 2019 May 15;10:1076. doi: 10.3389/fmicb.2019.01076 (PMC6530530; doi:10.3389/fmicb.2019.01076)
Supplement: FIGURE S2 — BLAST-P result using the AaHV1-encoded protein as a query. [file Image_2.pdf]

BLAST ® » blastp suite » RID-4UU7ESUN015

BLAST Results

Questions/comments

Job title: AaHV-FL

RID 4UU7ESUN015 (Expires on 01-28 15:16 pm)

Query ID lcl|Query_142472
Description AaHV-FL
Molecule type amino acid
Query Length 4228

Database Name nr
Description All non-redundant GenBank CDS translations+PDB+SwissProt+PIR+PRF excluding environmental samples from WGS projects
Program BLASTP 2.8.1+

New Analyze your query with SmartBLAST

Graphic Summary

Putative conserved domains have been detected, click on the image below for detailed results.

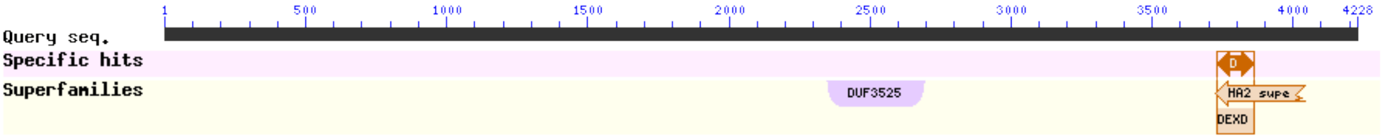

Distribution of the top 125 Blast Hits on 100 subject sequences

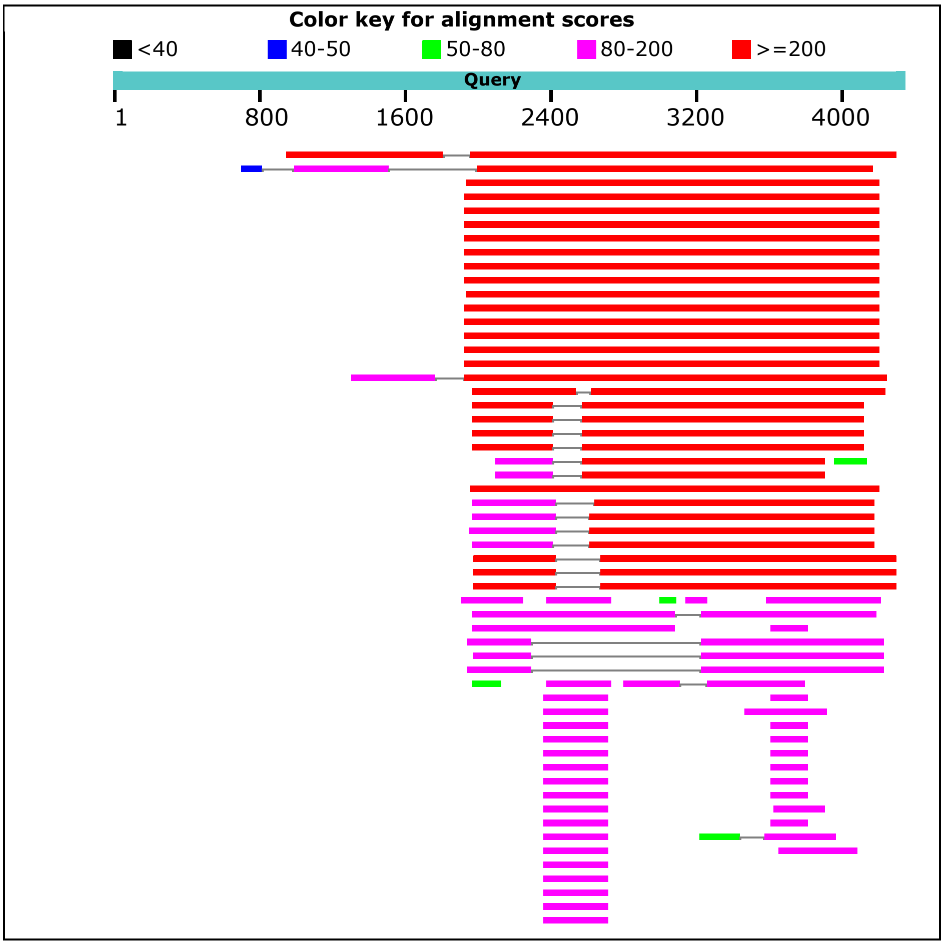

Descriptions

Sequences producing significant alignments:

Description	Max score	Total score	Query cover	E value	Ident	Accession
polyprotein [Wuhan insect virus 14]	2481	2782	74%	0.0	52%	YP_009342443.1
RNA-dependent RNA polymerase [Macrophomina phaseolina hypovirus 1]	1676	1878	64%	0.0	42%	ALD89099.1
ORF B [Cryphonectria hypovirus 1]	1475	1475	53%	0.0	38%	ATZ76099.1
ORF B [Cryphonectria hypovirus 1]	1474	1474	53%	0.0	38%	ATZ76097.1
ORF B [Cryphonectria hypovirus 1]	1474	1474	53%	0.0	38%	ATZ76095.1
ORF B [Cryphonectria hypovirus 1]	1474	1474	53%	0.0	38%	ATZ76109.1
hypothetical protein [Cryphonectria hypovirus 1]	1474	1474	53%	0.0	38%	NP_041091.1
RecName: Full=ORFB polyprotein; Contains: RecName: Full=Papain-like protease p48; Contains: RecName: Full=Putative RNA-directed RNA polymerase/helicase	1471	1471	53%	0.0	38%	Q9YTU2.1
ORF B [Cryphonectria hypovirus 1]	1471	1471	53%	0.0	38%	AUZ41744.1
ORF B [Cryphonectria hypovirus 1]	1471	1471	53%	0.0	38%	ATZ76103.1
ORF B [Cryphonectria hypovirus 1]	1470	1470	53%	0.0	38%	ATZ76111.1
polyprotein [Cryphonectria hypovirus 1]	1469	1469	53%	0.0	38%	AME30126.1
ORF B [Cryphonectria hypovirus 1]	1468	1468	53%	0.0	38%	ATZ76101.1
ORF B [Cryphonectria hypovirus 1]	1468	1468	53%	0.0	38%	ATZ76105.1
ORFB [Cryphonectria hypovirus 1]	1466	1466	53%	0.0	38%	ABI64296.1
ORF B [Cryphonectria hypovirus 1]	1459	1459	53%	0.0	38%	ATZ76107.1
hypothetical protein FgHV1gp2 [Fusarium graminearum hypovirus 1]	1396	1476	64%	0.0	35%	YP_009011065.1
polyprotein [Cryphonectria hypovirus 2]	1128	1521	50%	0.0	39%	NP_613266.1
replicase [Entoleuca hypovirus 1]	614	850	45%	1e-172	29%	AVD68669.1
polyprotein [Rosellinia necatrix hypovirus 2]	612	847	45%	6e-172	29%	BBB86776.1
polyprotein [Rosellinia necatrix hypovirus 2]	611	846	45%	2e-171	29%	BBB86794.1
replicase [Entoleuca hypovirus 1]	611	845	45%	2e-171	29%	AVK39795.1
polyprotein [Rosellinia necatrix hypovirus 2]	535	718	37%	4e-151	29%	BBB86803.1
polyprotein [Rosellinia necatrix hypovirus 2]	531	714	37%	4e-150	29%	BBB86801.1
polyprotein [Sclerotium rolfsii hypovirus 8]	528	528	52%	2e-146	25%	AZF86113.1
polyprotein [Rosellinia necatrix hypovirus 1]	515	690	46%	2e-142	28%	YP_009448196.1
polyprotein [Fusarium poae hypovirus 1]	504	680	46%	6e-139	28%	BAV56305.1
polyprotein [Fusarium graminearum hypovirus 2]	502	677	47%	2e-138	28%	YP_009130646.1
polyprotein [Fusarium langsethiae hypovirus 1]	501	669	46%	5e-138	28%	YP_009330037.1
replicase [Agaricus bisporus virus 2]	497	708	47%	9e-137	28%	AQM49947.1
replicase [Agaricus bisporus virus 2]	496	707	47%	2e-136	28%	AQM49946.1
replicase [Agaricus bisporus virus 2]	496	707	47%	2e-136	28%	AQM49918.1
hypothetical protein [Trichoderma hypovirus]	195	195	7%	5e-49	35%	AFR77082.1
polyprotein [Sclerotium rolfsii hypovirus 1]	190	376	47%	7e-44	24%	AZA15168.1
polyprotein [Sclerotium rolfsii hypovirus 1]	180	180	25%	3e-41	24%	AOX49921.1
polyprotein [Sclerotinia sclerotiorum hypovirus 2]	181	264	30%	5e-41	24%	YP_008828161.1
polyprotein [Sclerotinia sclerotiorum hypovirus 2]	181	261	30%	6e-41	24%	AIA61616.1
RNA-dependent RNA polymerase [Sclerotinia sclerotiorum hypovirus 2]	180	265	30%	9e-41	24%	AHE13861.1
ORF B [Cryphonectria hypovirus 1]	155	155	8%	6e-37	31%	AAP43896.1
ORF B [Cryphonectria hypovirus 1]	155	155	8%	1e-36	31%	AAP43901.1
ORF B [Cryphonectria hypovirus 1]	155	155	8%	1e-36	30%	AAP43895.1
ORF B [Cryphonectria hypovirus 1]	154	154	8%	3e-36	30%	AAO43503.1
ORF B [Cryphonectria hypovirus 1]	154	154	8%	3e-36	30%	AAP43900.1

ORF B [Cryphonectria hypovirus 1]	153	153	8%	4e-36	32%	AAO43502.1
OrfB [Cryphonectria hypovirus 1]	153	153	8%	6e-36	32%	AAM96812.1
ORF B [Cryphonectria hypovirus 1]	152	152	8%	9e-36	30%	AAP43898.1
OrfB [Cryphonectria hypovirus 1]	152	152	8%	9e-36	32%	AAM96807.1
OrfB [Cryphonectria hypovirus 1]	152	152	8%	1e-35	32%	AAM96815.1
OrfB [Cryphonectria hypovirus 1]	152	152	8%	1e-35	32%	AAM96804.1
OrfB [Cryphonectria hypovirus 1]	152	152	8%	1e-35	32%	AAM96789.1
OrfB [Cryphonectria hypovirus 1]	152	152	8%	1e-35	32%	AAM96809.1
OrfB [Cryphonectria hypovirus 1]	152	152	8%	1e-35	32%	AAM96800.1
OrfB [Cryphonectria hypovirus 1]	151	151	8%	3e-35	32%	AAM96817.1
OrfB [Cryphonectria hypovirus 1]	150	150	8%	3e-35	32%	AAM96814.1
OrfB [Cryphonectria hypovirus 1]	150	150	8%	3e-35	32%	AAM96801.1
OrfB [Cryphonectria hypovirus 1]	150	150	8%	4e-35	32%	AAM96803.1
OrfB [Cryphonectria hypovirus 1]	150	150	8%	4e-35	32%	AAM96802.1
OrfB [Cryphonectria hypovirus 1]	150	150	8%	4e-35	32%	AAM96794.1
OrfB [Cryphonectria hypovirus 1]	150	150	8%	5e-35	32%	AAM96799.1
OrfB [Cryphonectria hypovirus 1]	150	150	8%	6e-35	32%	AAM96797.1
ORF B [Cryphonectria hypovirus 1]	149	149	8%	1e-34	30%	AAP43894.1
polyprotein [Fusarium graminearum hypovirus 2]	154	154	14%	3e-33	25%	AKB94066.1
ORF B [Cryphonectria hypovirus 1]	144	144	8%	5e-33	29%	AAP43899.1
ORF2 [Ceratobasidium hypovirus A]	122	207	18%	1e-23	26%	AOX47536.1
replicase [Entoleuca hypovirus 1]	108	108	4%	4e-22	34%	AVK70320.1
replicase [Entoleuca hypovirus 1]	107	107	4%	6e-22	34%	AVK70315.1
ORF [Ceratobasidium hypovirus-like]	114	114	10%	7e-22	24%	AOX47561.1
replicase [Entoleuca hypovirus 1]	107	107	4%	8e-22	34%	AVK70309.1
replicase [Entoleuca hypovirus 1]	107	107	4%	9e-22	34%	AVK70314.1
replicase [Entoleuca hypovirus 1]	106	106	4%	1e-21	34%	AVK70307.1
replicase [Entoleuca hypovirus 1]	105	105	4%	5e-21	34%	AVK70313.1
replicase [Entoleuca hypovirus 1]	104	104	4%	6e-21	34%	AVK70318.1
replicase [Entoleuca hypovirus 1]	104	104	4%	7e-21	34%	AVK70325.1
putative RNA helicase [Agaricus bisporus virus X]	103	103	6%	1e-19	32%	CAD19173.1
replicase [Entoleuca hypovirus 1]	100	100	4%	3e-19	31%	AVK70326.1
ORF [Ceratobasidium hypovirus-like]	97.1	160	13%	8e-16	26%	AOX47559.1
polyprotein [Beihai sipunculid worm virus 6]	95.9	147	13%	3e-15	26%	YP_009333562.1
hypothetical protein BO78DRAFT_420497 [Aspergillus sclerotiicarbonarius CBS 121057]	84.3	84.3	2%	2e-12	39%	PYI04538.1
RNA-dependent RNA polymerase [Cryphonectria hypovirus 1]	72.4	72.4	1%	3e-11	46%	AZP89725.1
polyprotein [Sclerotinia sclerotiorum hypovirus 6]	80.9	80.9	9%	9e-11	25%	AZF86111.1
OrfB [Cryphonectria hypovirus 1]	71.6	71.6	4%	3e-09	30%	AZL49118.1
OrfB [Cryphonectria hypovirus 1]	71.2	71.2	4%	3e-09	27%	AZL49104.1
OrfB [Cryphonectria hypovirus 1]	71.2	71.2	4%	3e-09	29%	AZL49119.1
OrfB [Cryphonectria hypovirus 1]	71.2	71.2	4%	3e-09	29%	AZL49084.1
OrfB [Cryphonectria hypovirus 1]	71.2	71.2	4%	4e-09	29%	AZL49086.1
OrfB [Cryphonectria hypovirus 1]	70.9	70.9	4%	5e-09	29%	AZL49080.1
OrfB [Cryphonectria hypovirus 1]	70.9	70.9	4%	6e-09	29%	AZL49083.1
OrfB [Cryphonectria hypovirus 1]	70.5	70.5	4%	6e-09	29%	AZL49064.1
polyprotein [Rosellinia necatrix hypovirus 2]	74.7	74.7	3%	7e-09	33%	BBB86800.1
OrfB [Cryphonectria hypovirus 1]	70.1	70.1	4%	7e-09	28%	AZL49072.1
OrfB [Cryphonectria hypovirus 1]	70.1	70.1	4%	1e-08	29%	AZL49085.1
OrfB [Cryphonectria hypovirus 1]	70.1	70.1	4%	1e-08	28%	AZL49108.1
OrfB [Cryphonectria hypovirus 1]	70.1	70.1	4%	1e-08	29%	AZL49113.1
OrfB [Cryphonectria hypovirus 1]	70.1	70.1	4%	1e-08	29%	AZL49059.1

OrfB [Cryphonectria hypovirus 1]	69.7	69.7	4%	1e-08	29%	AZL49057.1
OrfB [Cryphonectria hypovirus 1]	69.7	69.7	4%	1e-08	27%	AZL49070.1
OrfB [Cryphonectria hypovirus 1]	69.7	69.7	4%	1e-08	29%	AZL49058.1
OrfB [Cryphonectria hypovirus 1]	69.3	69.3	4%	2e-08	28%	AZL49065.1
polyprotein [Rosellinia necatrix hypovirus 2]	72.8	72.8	3%	2e-08	31%	BBB86804.1
OrfB [Cryphonectria hypovirus 1]	69.3	69.3	4%	2e-08	29%	AZL49063.1
