# Supplementary material for: Identification of a Novel Hypovirulence-Inducing Hypovirus From Alternaria alternata
Source: Front Microbiol. 2019 May 15;10:1076. doi: 10.3389/fmicb.2019.01076 (PMC6530530; doi:10.3389/fmicb.2019.01076)
Supplement: TABLE S1 — A list of primers used in this study. [file Table_1.DOCX]

**Table S1. A list of primers used in this study.**

| Primer Name | Oligonucleotide sequence (5′- 3′) | Usage |
| --- | --- | --- |
| ITS1 | TCCTCCGCTTATTGATATG | For Ribosomal RNA ITS region of *Alternaria alternate* f. sp. *mali* |
| ITS2 | GGAAGTAAAAGTCGTAACAAGG |  |
| 3C-520R | ACGCTTTGACGTCGGGAATACGCTC | For 5’ RACE of AaHV  For 3’ RACE of AaHV |
| 3C-13750F | TCGAGGAGACGGGCGGCACTGG |  |
| 3C-180F | ACGCTATGCGTGAGCCCACCAAG | For AaHV1 Northern blot probe N |
| 3C-900R | TGAACATGCGGCGCAACAGTGATG |  |
| 3C-13486F | AACGGAACCTGAAAATGATCTCG | For AaHV1 Northern blot probe C |
| 3C-14039R | TGATACATCAGGCACACCGCAGTG |  |
| 3C-7981F | TTGCAGCGGGAATTGAAGGCATACC | For AaHV1 Northern blot probe M |
| 3C-8603R | AATTTCAGCAGCCAGTCTAGCCC |  |
| 3C- C1F-12735F | ATCCATGCCCTTTCATTATACG | For AaHV1 Genomic and D-RNA RT-PCR sequencing |
| 3C-C1R-13476R | AGGTTCCGTTGTCTCGTCAC |  |
| 3C- C2F-12086F | GCTTGAGCCGAATATTCAAAGAC |  |
| 3C- C2R-12816R | GTCAGTCCGATGTAATCATCTCC |  |
| 3C-C3F-11436F | CCTCGGGACCCATATCTATATTC |  |
| 3C-C3R-12136R | TCTGCCATACCCATAGAATCAG |  |
| 3C-C4F-10756F | AACTTAGCCCTCGACAGGTG |  |
| 3C-C4R-11525R | GCAGCATATCGATGACGAGAATG |  |
| 3C-C5F-10021 F | CAAACACCCATGCCAAAGATAG |  |
| 3C-C5R-10778R | CTTTCAGCCACTCGAGGAAG |  |
| 3C-C6F-9326F | CCAGGATCAAGCCGTACAAATAG |  |
| 3C-C6R-10033R | GTGCTACCTGCACTATCTTTGG |  |
| 3C-C7F-8597F | TGAAATTGCCTACACGGAGTTC |  |
| 3C-C7R-9362R | CCGTATTCCTGCCAAGTGATG |  |
| 3C-C8F-7897F | CGGAAGTTCTGCTCAAGATCATAC |  |
| 3C-C8R-8651R | GATTGGCTTGACTGACTAGTTCC |  |
| 3C-C9F-7176F | CCAAAGGTCGTCCAATGGTATG |  |
| 3C-C9R-7898R | CGTATGATCTTGAGCAGAACTTCC |  |
| 3C-C10F-6504F | AAGAATTTCACAGTCCCAGAGC |  |
| 3C-C10R-7182R | AACGCATACCATTGGACGAC |  |
| 3C-C11F-5771F | ACGAGAAGACTATGCCATGC |  |
| 3C-C11R-6532R | CGTTAAGCTCTGGGACTGTG |  |
| 3C-C12F-5079F | AAGTCGCTGGACCCAAATTC |  |
| 3C-C12R-5836R | GTGCTGCTACGGAATTTCTTG |  |
| 3CC13F-4360F | CAAGGAGAGTGTCGAAAGAAG |  |
| 3C-C13R-5092R | CAACACCTTTGCGGAATTTG |  |
| 3C-C14F-3674F | CTTGGCACTCAGAAATTACC |  |
| 3C-C14R-4391R | CACGCAAATCCTCATTCATC |  |
| 3C-C15F-2988F | AGTCTTTGGGATCTGAAGTC |  |
| 3C-C15R-3755R | CAGGATAAACGCATGGTATG |  |
| 3C-C16F-2291F | CATGATGGTGTTGAAGGAAAG |  |
| 3C-980F | AGGGATGGAGAGATAGTGGATAACGG |  |
| 3C-6838R | AGTCCGTCAGCTGATCTCGGAAG |  |
| 3C-1390F | ACCACGAATTCTAGCGCCTGTCAG |  |
| 3C-3284R | TACGCCTATGGACCCGTCGCCTG |  |
| 3C-1891F | CAACTTCATCTCCGCTGCGATAAGG |  |
| 3C-2620R | GTGACGGCAAGGATAGGTGATGCTC |  |
| 3C-3320F | AGGACAGGGCATGTGCAATCAAG |  |
| 3C-3920F | AGGATAGGTTCTGGTCAACTAATGGG |  |
| 3C-4840F | AGTGAGTCACCCATCGACCCGGATC |  |
| 3C-5680F | AGAGGCTCTGGTGGTGCATTGAGGG |  |
| 3C-6180F | GTTCGACTAAACCGGCCGTGAATG |  |
| BdITS1 | TCCGTAGGTGAACCTGCGG | For Ribosomal RNA ITS region of *Botryosphaeria dothidea* |
| BdITS4 | TCCTCCGCTTATTGATATGC |  |
| 3C-4344F | ACCTTCCCTGACTGGCCAAGGAG | For detection of D-RNA by RT-PCR using specific primer designed from junction region, 418 bp |
| D-RNA4742R | ACCACGAGGCAATACAAAGGC |  |
