# Supplementary material for: Identification of a Novel Hypovirulence-Inducing Hypovirus From Alternaria alternata
Source: Front Microbiol. 2019 May 15;10:1076. doi: 10.3389/fmicb.2019.01076 (PMC6530530; doi:10.3389/fmicb.2019.01076)
Supplement: TABLE S2 — Detection of AaHV1 in 43 Alternaria alternata strains. [file Table_2.DOCX]

**Table S2. Detection of AaHV1 in 43 *A. alternata* strains.**

| Strain name  A. alternate- | dsRNA detection | AaHV1 infection  Detected by RT-PCR |
| --- | --- | --- |
| YL-6D | **Positive** | **Not detected** |
| YL-5D1 | **Positive** | **Not detected** |
| YL-4A | **Not detected** | **Not detected** |
| YL-5D2 | **Not stable** | **Not detected** |
| YL-4D | **Positive** | **Not detected** |
| YL-A3 | **Not detected** | **Not conducted** |
| YL-1D | **Positive** | **Not detected** |
| YL-3C | **Positive** | **Yes** |
| YL-2C | **Not stable** | **Yes/Not stable** |
| YL-1A | **Not detected** | **Not detected** |
| YL-1P | **Not detected** | **Not detected** |
| YL-2B | **Positive** | **Not detected** |
| YL-2P | **Not detected** | **Not detected** |
| YL-2D | **Positive** | **Not detected** |
| YL-2A | **Not detected** | **Not detected** |
| YL-3D | **Positive** | **Not detected** |
| YL-1F | **Positive** | **Not detected** |
| YL-2P | **Not detected** | **Not detected** |
| YL-7D | **Positive** | **Not conducted** |
| YL-3P | **Not detected** | **Not detected** |
| YL-4P | **Not detected** | **Not detected** |
| YL-5P | **Positive** | **Not detected** |
| YL-8D | **Positive** | **Not detected** |
| YL-1G | **Positive** | **Not detected** |
| YL-6P | **Not detected** | **Not detected** |
| YL-7P | **Not detected** | **Not conducted** |
| YL-8P | **Not detected** | **Not conducted** |
| YL-9P | **Not detected** | **Not detected** |
| YL-9D | **Positive** | **Not conducted** |
| YL-10P | **Not detected** | **Not conducted** |
| YL-11P | **Not detected** | **Not detected** |
| YL-1E | **Positive** | **Yes** |
| YL-12P | **Not detected** | **Not conducted** |
| YL-15P | **Not detected** | **Not detected** |
| YL-18P | **Not detected** | **Not conducted** |
| YL-19P | **Not detected** | **Not conducted** |
| YL-20P | **Not detected** | **Not conducted** |
| YL-21P | **Not detected** | **Not conducted** |
| YL-22P | **Not detected** | **Not detected** |
| YL-23P | **Not detected** | **Not detected** |
| YL-24P | **Not detected** | **Not conducted** |
| YL-25P | **Not detected** | **Not detected** |
| YL-26P | **Not detected** | **Not conducted** |
